# Interleukin-21 engineering enhances CD19-specific CAR-NK cell activity against B-cell lymphoma via enriched metabolic pathways

**DOI:** 10.1186/s40164-025-00639-2

**Published:** 2025-04-02

**Authors:** Bailin He, Hong Chen, Jiaxu Wu, Shiqiu Qiu, Qiusui Mai, Qing Zeng, Cong Wang, Shikai Deng, Zihong Cai, Xiaoli Liu, Li Xuan, Chengyao Li, Hongsheng Zhou, Qifa Liu, Na Xu

**Affiliations:** 1 Department of Hematology, Nanfang Hospital, Southern Medical University, Guangzhou, China; 2 Guangdong Provincial Clinical Research Center for Hematologic Diseases, Nanfang Hospital, Southern Medical University, Guangzhou, China; 3 Department of Transfusion Medicine, School of Laboratory Medicine and Biotechnology, Southern Medical University, Guangzhou, China; 4 Department of Hematology, The First Affiliated Hospital of Guangdong Pharmaceutical University, Guangzhou, China; 5 Department of Blood and Transfusion, The Seventh Affiliated Hospital, Sun Yat-Sen University, Shenzhen, China

**Keywords:** Chimeric antigen receptor (CAR)-NK cells, Interleukin-15, Interleukin-21, B-cell malignances, Metabolic fitness

## Abstract

**Background:**

NK cells engineered to express interleukin-15 (IL-15) and a CD19-targeted chimeric antigen receptor (CAR) have been used to treat patients with relapsed and/or refractory B cell malignances, demonstrating encouraging outcomes and favorable safety profile. However, the effect of IL-21 in CAR-NK cell therapy remains unknown.

**Methods:**

CD19-specific CAR with 4-1BB costimulatory domain and cytokine IL-21 or IL-15 was constructed and transduced into peripheral blood (PB)-derived NK cells to produce CD19-CAR-IL21 NK cells (CAR-21) or CD19-CAR-IL15 NK cells (CAR-15), respectively. The phenotypic profile, transcriptomic characteristics, functionality and anti-tumor activity of CAR-21 NK cells and CAR-15 NK cells were compared.

**Results:**

Compared with CAR-NK cells co-expressing IL-15, CAR-NK cells co-expressing IL-21 exhibited significantly increased IFN-γ, TNF-α and Granzyme B production, as well as degranulation, in response to CD19^+^ Raji lymphoma cells, resulting in enhanced cytotoxic activity upon repetitive tumor stimulation. Furthermore, IL-21 co-expression improved the in vivo persistence of CAR-NK cells and significantly suppressed tumor growth in a xenograft Raji lymphoma murine model, leading to prolonged survival of CD19^+^ tumor-bearing mice. RNA sequencing revealed that CAR-21 NK cells have a distinct transcriptomic signature characterized by enriched in cytokine, cytotoxicity, and metabolic related signaling, when compared with CAR-15 NK or CAR NK cells.

**Conclusions:**

This study demonstrated that CD19-specific CAR-NK cells engineered to express IL-21 exhibit superior persistence and anti-tumor activity against CD19^+^ tumor compared to CAR-NK cells co-expressing IL-15, which might be a promising therapeutic strategy for treating patients with relapse or refractory B cell malignances.

**Supplementary Information:**

The online version contains supplementary material available at 10.1186/s40164-025-00639-2.

## Introduction

Chimeric antigen receptor (CAR)-T [1] cell therapies have received approval from the Food and Drug Administration (FDA) for the treatment of B cell malignancies and multiple myeloma [2, 3]. Despite the impressive successes of CAR-T cells, limitations such as high cost, length of manufacturing, and CAR-T cell related toxicities like cytokines release syndrome (CRS) and immune effector cell-associated neurotoxicity syndrome (ICANS) have led to growing interest in exploring alternative approaches [4]. Natural killer (NK) cells present a promising alternative to T cells for cancer immunotherapy. NK cells recognize their targets in a major histocompatibility complex-unrestricted manner, implying a low risk of graft-versus-host disease (GVHD) during allogeneic transfusion [5–7]. In clinical studies, CAR-NK cells have demonstrated efficacy in treating patients with hematological malignances without causing noticeable toxicity or CRS [8–10]. These unique features provide NK cells with significant advantages as an off-the-shelf product for immediate clinical use. Nevertheless, poor post-infusion persistence of NK cells remains a major challenge for CAR-NK cell therapy.

NK cells require signaling through cytokine receptors to promote their survival, expansion, and persistence [11]. Common cytokine receptor γ chain family cytokines, such as IL-2, IL-15, and IL-21, play fundamental roles in NK cell development, differentiation, and effector phases [12]. Systemic IL-2 administration can cause severe toxicities like vascular leak syndrome and lead to the activation of regulatory T cells, which might impair NK cell function [13–15]. Systemic IL-15 administration stimulates recipient CD8^+^ T cell activation, which may accelerate donor NK cell rejection and limit allogeneic cellular therapy [16–18]. IL-21, a pleiotropic cytokine influencing various immune cell types, is known to promote NK cell proliferation, maturation, and metabolic fitness [19–22]. Recombinant IL-21 has been tested in several clinical trials for metastatic cancer and has shown a desirable safety profile [23, 24]. Recent studies by Shanley et al. demonstrated that locoregionally administered NK cells engineered to express IL-21 show superior safety and long-term anti-tumor activity compared to those engineered to express IL-15 [25]. However, the safety and efficacy of IL-21 in CAR-NK cell-mediated tumor rejection are lacking.

IL-15 and IL-21 promote NK cell expansion and survival, possessing both overlapping and distinctive functions [21]. In this study, we generated IL-15 or IL-21-secreting CD19-specific CAR-NK cells to compare their functional and phenotypic characteristics. CAR-21 NK cells showed similar immunophenotypic profiles compared to CAR-15 NK cells, while IL-21 engineered CAR-NK cells exhibited significantly increased persistence and superior anti-tumor efficacy against CD19^+^ Raji lymphoma cells both in vitro and in vivo. By comparing the transcriptional profiles of CAR-15 and CAR-21 NK cells, we found that the anti-tumor effect can be enhanced by armoring CAR-NK cells with IL-21 to increase their metabolic fitness and effector function. These data support the use of IL-21 armored CAR-NK cells as a potential immunotherapeutic approach for treating patients with relapse or refractory CD19^+^ B cell malignancies.

## Methods

### Ethical statement

Human peripheral blood mononuclear cells (PBMCs) from healthy human donors were obtained for this study following informed consent. All animal studies were approved by the Institutional Animal Care and Use Committee of Southern Medical University. The study received approval from the Ethics Committee of Southern Medical University (No. SMUL2023080) and the Guangzhou Blood Center (No. GZBC2022068).

### Generation of CD19-specific CAR-NK cells with IL-15 or IL-21 co-expression

**Fig. 1:**
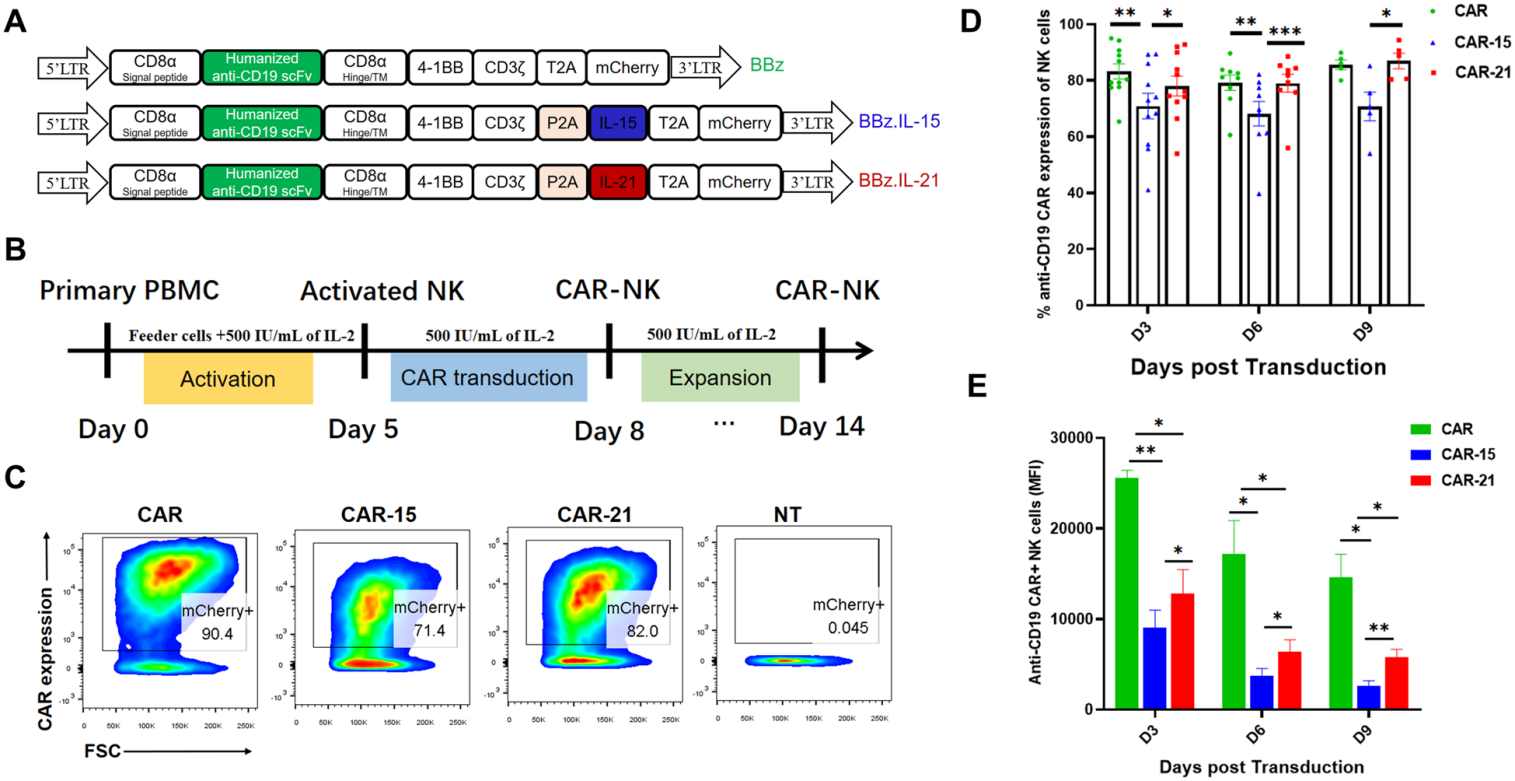
Construction of CD19-targeting CAR and characteristics of anti-CD19 CAR-NK cells. **A **Schematic diagram of the BBz, BBz.IL-15, and BBz.IL-21constructs. **B** Schematic diagram of generation of CAR-NK cells. Peripheral blood mononuclear cell (PBMC) was stimulated with irradiated engineered K562 feeder cells on day 0 and transduced with retroviral vectors on day 5. NK cells were cultured for a total of 14 days to allow for expansion. **C** Representative flow cytometric plots of CAR, CAR-15 and CAR-21 NK cells and non-transduced (NT)-NK cells labeled by mCherry. **D** Statistic graph of transduction efficiency of CAR, CAR-15 and CAR-21 on days 3, 6 and 9 post transduction (n = 11 for days 3 and 6; n = 4 for day 9). **E** Statistic graph of mCherry MFI of CAR, CAR-15 and CAR-21 on days 3, 6 and 9 post transduction (n = 11 for days 3 and 6; n = 4 for day 9).

Firstly, a humanized CD19-targeting CAR (hCD19-CAR) construct was created by cloning the humanized single-chain fragment variable (scFv) of the mouse monoclonal antibodies FMC63. This was achieved by grafting the complementarity-determining regions (CDRs) onto the human frameworks IGHV4-59*01 (VH) and IGKV1-39*01 (VL) and integrating them in-frame into a second-generation CAR backbone. The codon-optimized minigene encoding cytokine IL-21 or IL-15, linked to hCD19-CAR containing 4-1BB costimulatory domain via a P2A sequence, was synthesized and sub-cloned into the pSFG retroviral vector, yielding the BBz.IL-21 or BBz.IL-15 retroviral construct (Fig. 1A). Recombinant retroviruses were produced by co-transfecting retroviral plasmids and packing plasmids (PegPam3 plasmid and RDF plasmid) into HEK-293 T/17 cells as previous described [26, 27]. Human PBMCs were isolated by density gradient centrifugation from the “Buffy Coat” of healthy blood donors at the Guangzhou Blood Centre using Human Peripheral Blood Lymphocyte Separation Solution (Tianjin Haoyang Biological Manufacture Co., Ltd, China). To activate human NK cells, the isolated PBMCs were stimulated with irradiated (10 Gy) K562-4-1BBL-mIL-15 at a 1:5 feeder cell to PBMC ratio for 5 days in NK cell culture medium (ExCell Biology, Inc., Shanghai, China) supplemented with 500 IU/mL recombinant human IL-2 (PeproTech). CD56^+^ NK cells, purified with an NK isolation kit (Miltenyi Biotec, Inc., San Diego, CA, USA) were then transduced with recombinant retrovirus on RetroNectin® (T100A, TaKaRa)-coated plates according to the manufacturer’s instructions (Takara, Japan). The recombinant BBz, BBz.IL-15 or BBz.IL-21 retrovirus-transduced NK cells were designated as CAR, CAR-15 or CAR-21 NK cells, respectively, while the non-transduced (NT)-NK cells served as controls. The expression of CD19-specific CAR was analyzed by flow cytometry for red fluorescent protein (mCherry). Cell counts were recorded over time during media changes to monitor the proliferative capacity of NK cells.

### Degranulation assay

CAR, CAR-15 and CAR-21 NK cells was co-cultured with Raji target cells in a medium containing protein transport inhibitor (brefeldin A and monensin) and CD107a (BioLegend) antibody at 37 ℃ with 5% CO_2_ for 4 h. The cells were then washed and stained with CD3 and CD56, the surface expression of CD107a in the CAR^+^ NK cells was determined by flow cytometry.

### Intracellular cytokine and transcription factor staining

In the cytokine release analysis, CAR, CAR-15 and CAR-21 NK cells were stimulated with Raji cells (E: T = 2.5 × 10^5^ NK cells/1 × 10^5^ Raji) for 4 h in the presence of monensin and brefeldin A. For the transcription factor expression analysis, CAR, CAR-15 and CAR-21 NK cells were stimulated with or without Raji cells (E: T = 2.5 × 10^5^ NK cells/1 × 10^5^Raji) for 4 h. After the incubation, cells were harvested and stained with Fluorochrome-labeled monoclonal antibodies: LIVE/DEAD Fixable Dead Cell Staining, CD3, and CD56 for 20 min at room temperature. After cell fixation and permeabilization with the BD Fixation/Permeabilization Kit, cells were stained with interferon γ (IFN-γ), Tumor Necrosis Factor α (TNF-α) and Granzyme B for 30 min or with EOMES, T-bet and GATA3 for 50 min on ice in the dark. After washing once with 1 mL of FACS buffer, cells were re-suspended in 200 µL FACS buffer for acquisition.

### Luciferase-based cytotoxicity assays

To assess the cytotoxic capacity of NK cells, a firefly luciferase reporter was introduced into target cells (CD19^+^ Raji and CD19^−^K562) and detected using the substrate D-luciferin to measure changes. NK cells (CAR, CAR-15, CAR-21 and NT NK cells) were harvest on day 7 post transduction and co-cultured with Raji-FFLuc or K562-FFLuc at Effector to Target (E: T) ratios of 5:1, 2.5:1, 1.25:1, 0.625:1 for 8 h (n = 4). Luminescence intensity was measured using a luminescence microplate reader after the addition of the D-Luciferin substrate. The luminescence readings were expressed as Relative Light Unit (RLU). The percentage of tumor cell killing by NK cells was calculated using the following equation: % Target Lysis = 100% × (RLU of untreated tumor cells–RLU of tumor cells cultured with NK cells)/RLU of untreated tumor cells.

### In vitro repeated co-culture assay

CAR^+^ NK cells (1 × 10^5^) were plated in triplicate wells of 96-well plates using NK cell medium without cytokine. Raji-GL tumor cells (1 × 10^5^) were then added to each well (round 1). Every 12 h, half of the supernatant in each well was gently removed, and an equivalent volume of Raji-GL cells (1 × 10^5^) in fresh NK cell medium was added. This process was repeated for two additional rounds (round 2 and 3). At the end of each round, Raji-GL cells in duplicate wells were collected to determine the absolute number of Raji-GL cells (GFP^+^) in the co-culture by CountBright Absolute Counting Beads (Thermo Fisher Scientific) via flow cytometry (n = 4).

### Seahorse assay

CAR-NK cells were assayed alone or purified after 24 h co-culture with Raji cells. Cells were resuspended in Seahorse XF RPMI medium (Agilent) supplemented with 2 mM l-glutamine, 1 mM sodium pyruvate and 10 mM D-glucose and seeded at 200,000 cells per well in a 96 well microplate. XFe96 cell culture microplates (Agilent) were pre-coated with Poly-d-Lysine (Sigma) over-night at 37 °C. The extracellular acidification rate (ECAR) and oxygen consumption rate (OCR) were measured (pmol/min) in real time in an Agilent Seahorse XFe96 Analyzer using Seahorse XF Glycolytic Rate Assay Kit (Agilent) and Seahorse XF Cell Mito Stress Test Kit (Agilent) per the manufacturer’s instructions. Data were analyzed using Wave Software (Agilent).

### Animal experiment

Six-to eight-week-old female NOD-SCID-γ (NSG) mice were intravenously (IV) inoculated with Raji-GL cells (5 × 10^5^) (Day 0). Four days later, mice with growing xenografts were randomized to receive a single dose of 5 × 10^6^ total NK cells in 200 µL of PBS containing 85% to 95% CAR^+^ cells (Supplemental Fig. 1) or 200μL of PBS. Lymphoma growth was monitored in vivo over time by quantifying photon emission using the Xenogen In vivo Imaging System (IVIS, Bruker) after intraperitoneal injection of d-luciferin salt (3 mg per 200 μL) and tracked for survival. The body weight of the mice was measured every 2 to 5 days. Once the mice exhibited bilateral hind limb paralysis or weight loss exceeding 20% of their initial body weight, they were anesthetized, and organs were collected and assessed for the presence of NK cells and GFP^+^ Raji cells.

### Statistical analysis

All data were analyzed with GraphPad Prism version 8. Data are presented as the mean ± standard error of the mean (SEM). Between-group differences (mean ± SEM) were compared using the paired Student* t*-test, two-sample Student *t*-test, or analysis of variance (ANOVA) as appropriate. *P* values were two-sided, and *P* values < 0.05 were considered significant. Kaplan–Meier survival curves from animal experiments were analyzed using the log-rank test. Statistical differences are indicated by asterisks: ****P < 0.0001; ***P < 0.001; **P < 0.01; *P < 0.05; ns indicates not significant.

Additional detailed methods are provided in supplemental Data.

## Results

### Generation of IL-21-expressing CD19-specific CAR-NK cells

CD19-specific CAR-NK cells were prepared using human PBMC-derived NK cells and transduced with CD19.CAR retroviral vectors with or without IL-15 or IL-21 (Fig. 1A, B). CAR expression by NK cells was measured by expression of mCherry in these CAR constructs. The transduction efficiency reached to 83.21 ± 2.66% in CAR NK cell group, 70.93 ± 4.59% in CAR-15 NK cell group and 78.04 ± 3.55% in CAR-21 NK cell group on Day 3 post transduction (Fig. 1C, D). The transduction efficiency of the three types of CAR-NK cell groups remained stable on day 6 and day 9 post transduction (Fig. 1D), but the median fluorescence intensity (MFI) of CAR expression continued to decrease (Fig. 1E). On day 3, 6 and 9 post transduction, the transduction efficiency of CAR-21 NK cells was comparable to that of CAR NK cells but significantly higher than that of CAR-15 NK cells. Moreover, CAR-21 NK cells were associated with higher CAR intensity of expression when compared with CAR-15 NK cells. Taken together, we successfully generated IL-21 co-expressing CD19-specific CAR-NK cells with high transduction efficiency.

### IL-21 secretion promoted the survival of CAR-NK cells

**Fig. 2:**
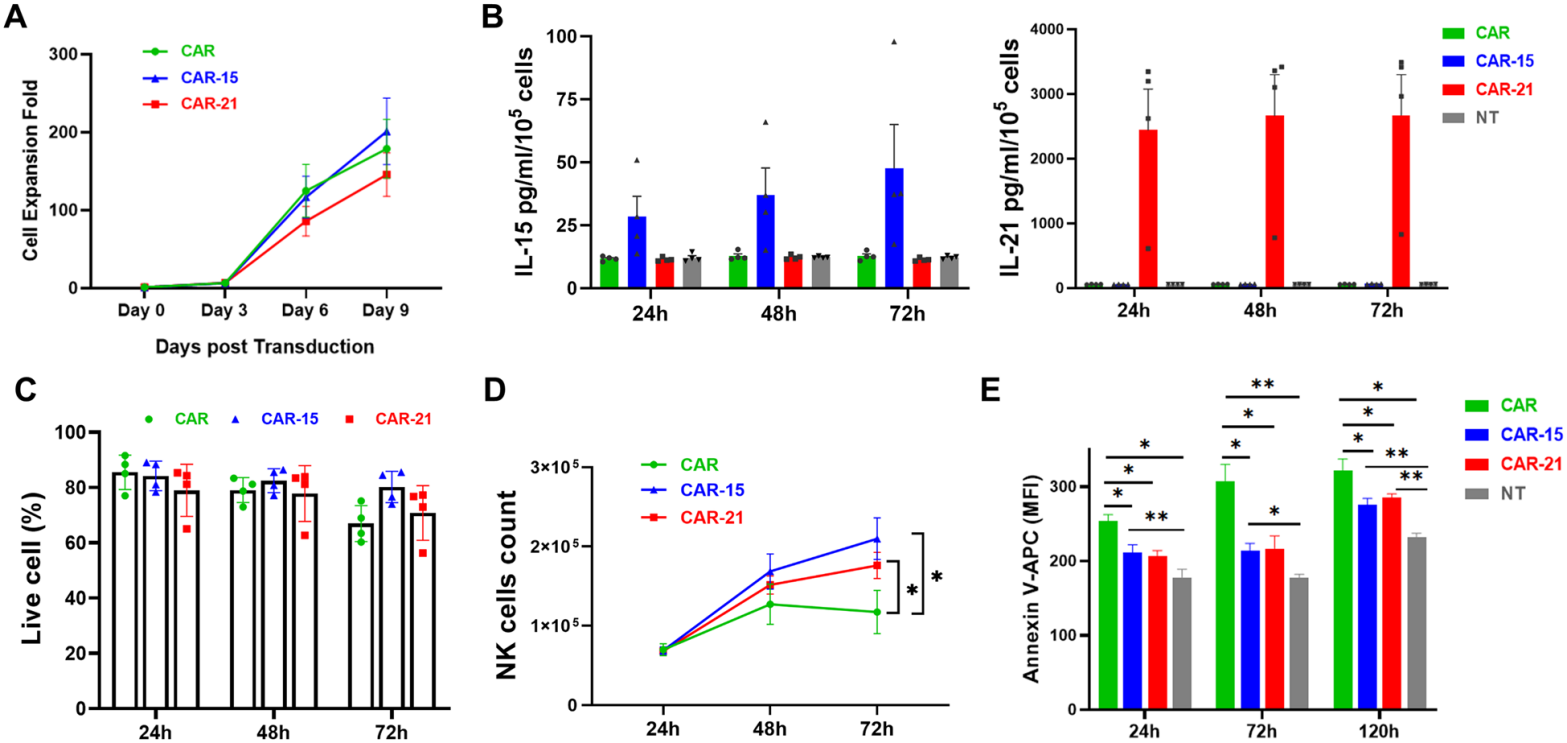
Proliferation and survival of CD19-specific CAR-NK cells with IL-21 expression. **A** Proliferation multiplicity of CAR, CAR-15 and CAR-21 NK cells in culturing with IL-2 maintenance (n = 4 donors). **B** IL-15 and IL-21 levels by ELISA from supernatants from non-transduced (NT), CAR, CAR-15 or CAR-21 NK cells (n = 4 donors). **C** The percentage of viable NK cells in cultures without IL-2 maintenance (n = 4 donors). **D** The numbers of viable NK cells in cultures without IL-2 maintenance (n = 4 donors). **E** The percentage of Annexin V positive NK cells in cultures without IL-2 maintenance (n = 4 donors).

By conventionally NK cell culturing with IL-2, the growth curve (fold change) showed that CAR, CAR-15 and CAR-21 NK cell groups proliferated 200-folds approximately after nine days post transduction, but no significant difference between the three groups (*P* > 0.05) (Fig. 2A). Mimicking in vivo situation without IL-2 maintenance, 0.1 million NT-NK, CAR, CAR-15 and CAR-21 NK cells were harvest on Day 5 post transduction and cultured in a 96 well plate. High levels of IL-21 were found in the supernatant of CAR-21 NK cells after resting 24 h (2445 ± 630.50 pg/mL), while the lower concentration (about 60 pg/mL) was detected in those of NT, CAR and CAR-15 NK cell groups; CAR-15 NK cells (28.5 ± 8.06 pg/mL) produced significant more IL-15 than NT, CAR and CAR-21 NK cells (about 10 pg/mL) after resting 24 h (Fig. 2B). The concentration of IL-21 and IL-15 in culture supernatant of each group remained stable over the subsequent 48 and 72 h. After culturing 24 ~ 72 h, cell viability among CAR NK cell group was declined from 85 to 70%, whereas cell viability among CAR-15 and CAR-21 NK cell groups still maintained 80% viable cells (Fig. 2C). The live cell counts of three groups were determined using the Flow cytometry Absolute Counting Beads simultaneously (Fig. 2D), in which the cell counts of the three groups increased in 24 ~ 48 h, with the CAR-15 and CAR-21 NK cell groups continued to increase in 48 ~ 72 h, while CAR NK cell group decreased. Both IL-15 or IL-21 overexpressed CAR-NK cells exhibited higher proliferation capacity, but there was no significant difference between the two groups. Meanwhile, we examined whether the observed differences in proliferation were related to differences in apoptosis using Annexin V staining. We found that co-expression of IL-15 or IL-21 decreased the apoptosis in NK cells resulting in more live cells compared with CAR NK alone as measured at 24, 72 and 120 h cultured without IL-2 maintenance (Fig. 2E). Together, CAR-21 NK cells produced high levels of IL-21 which promoted the survival of CAR-NK cells during at rest.

### Immunophenotypic analysis of CAR-NK cells co-expressing IL-21

**Fig. 3:**
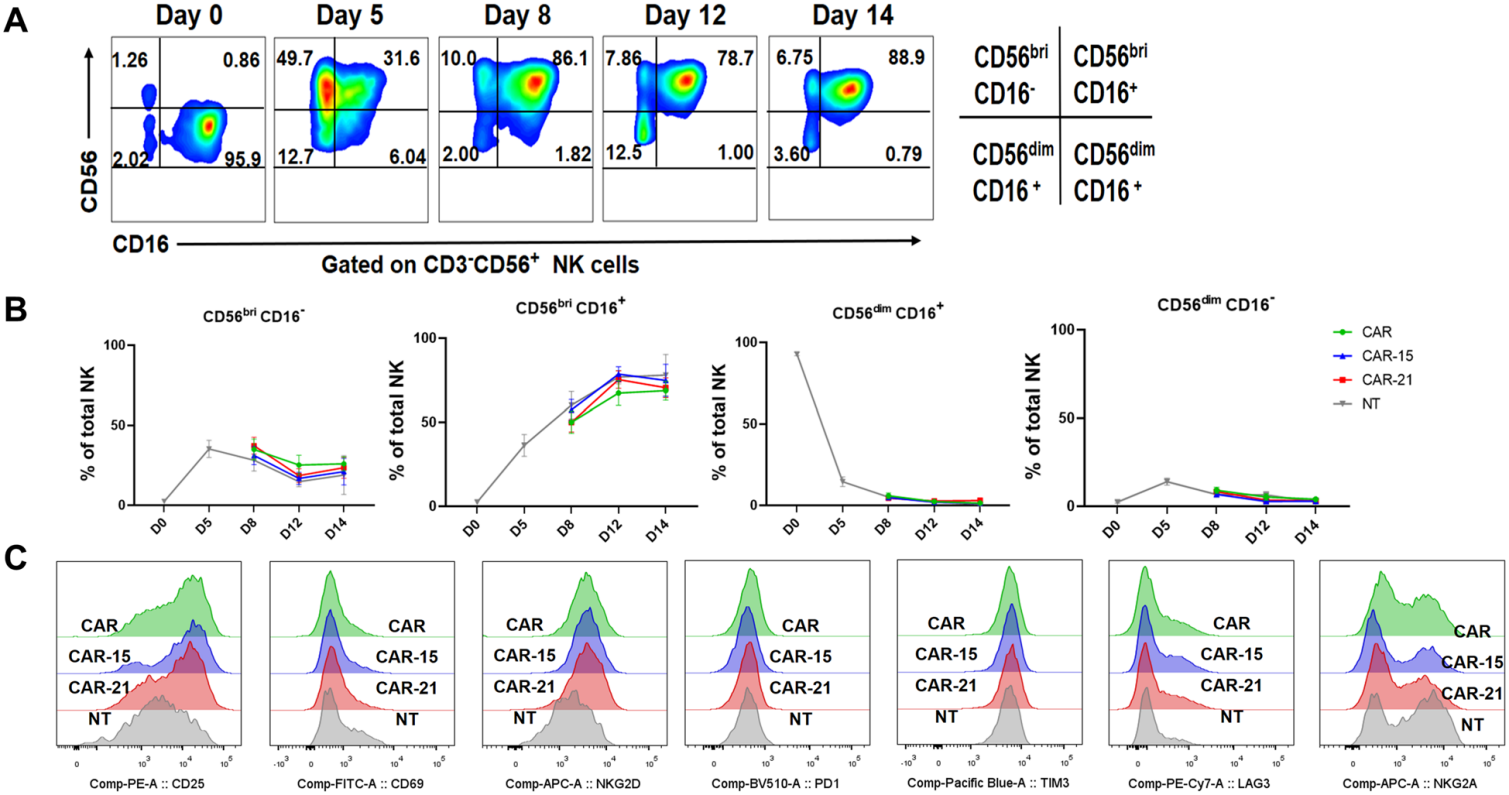
Phenotype signatures of CAR-NK cells co-expressing IL-21. **A** Representative flow cytometry plots showing the distribution of NK cell subtypes based on the relative expression of CD56 and CD16. **B** Evaluation of NK cell subsets over time in NT, CAR, CAR-15 and CAR-21 NK cell groups (n = 11 for days 3 and 6; n = 4 for day 9). **C** Representative flow cytometric histograms of activation (CD25, CD69, NKG2D), inhibition (NKG2A), and exhausted (PD-1, Tim-3, LAG-3) markers expression in CD3^−^ CD56^+^ NT, CAR, CAR-15 and CAR-21 NK cells after CAR transduction.

Next, the subtypes and functional biomarkers of CAR-NK cells were analyzed. Four NK cell subsets including CD56^bright^CD16^+^, CD56^bright^CD16^−^, CD56^dim^CD16^+^, CD56^dim^CD16^−^ cells were found during the generation and expansion of CAR-NK cells (Fig. 3A). The population of CD56^bright^CD16^+^ cells was low before K562 feeder cells activation but constituted the majority population in NK cells on day 14 (Fig. 3B). In contrast, CD56^dim^CD16^+^ NK subset, which is responsible for natural cytotoxicity and constitute majority of NK cells in peripheral blood, were continuously decreased during ex vivo culture (Fig. 3B). A similar trend of the evolution of NK cell subsets was seen in CAR, CAR-15 and CAR-21 NK cells (Fig. 3B). To determine the phenotypic profiles of CAR-NK cells, we used flow cytometry to characterize several important activating and inhibitory receptors, as well as exhaustion markers (Fig. 3C). The findings revealed an enhanced expression of CD25 and NKG2D in CAR-NK cells compared to NT-NK cells, but no statistically significant difference between CAR, CAR-15 and CAR-21 NK cell groups (*P* > 0.05). In addition, expression of activation marker CD69, inhibitor receptor NKG2A, as well as exhaustion markers PD1, TIM3 and LAG3 were comparable between CAR-NK cells and NT-NK cells. Therefore, CAR-21 NK cells displayed a similar immunophenotypic profiles to CAR-15 NK and CAR NK cells.

### Transcriptomic signatures of CAR-NK cells co-expressing IL-21

**Fig. 4:**
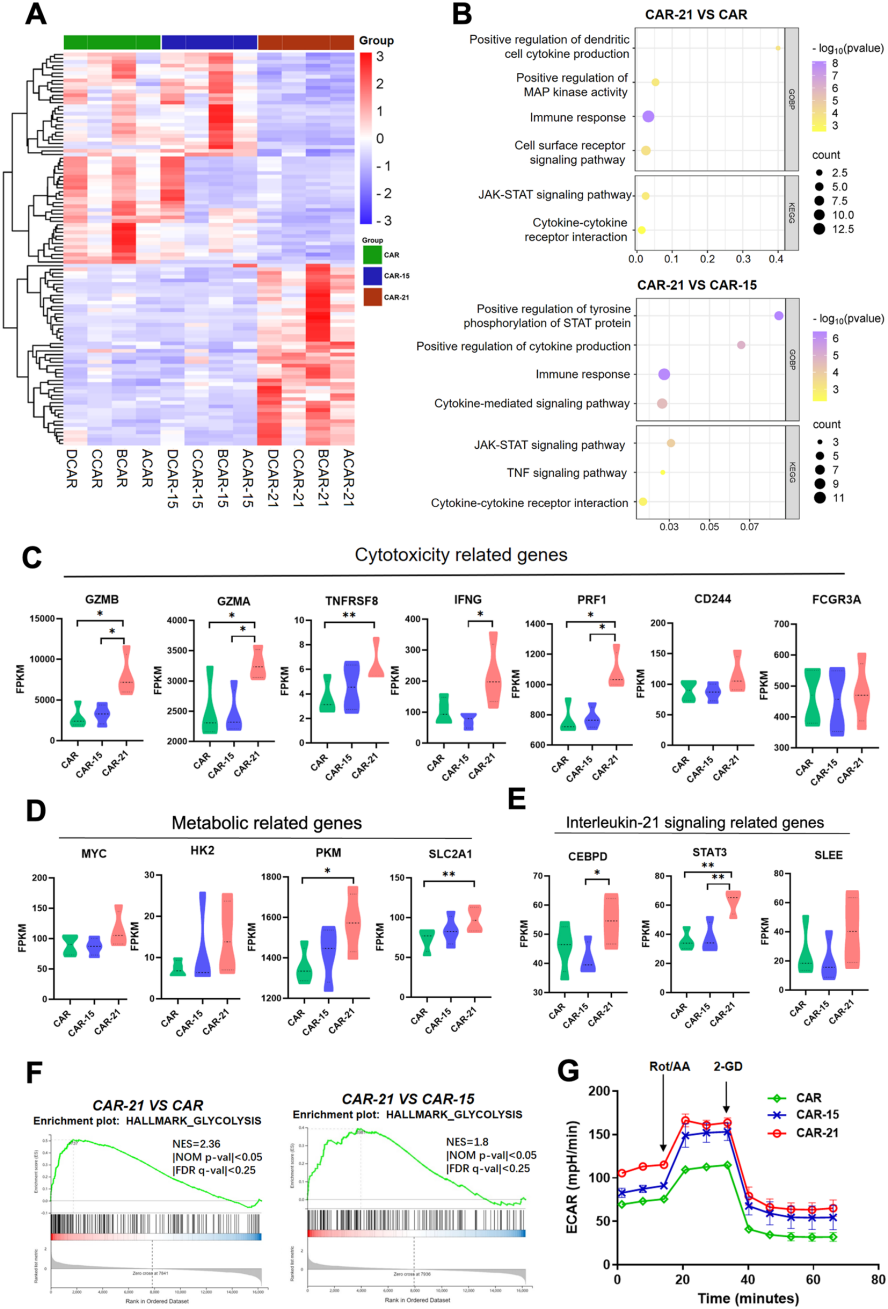
Transcriptomic signatures of CAR-NK cells co-expressing IL-21. **A** Heatmap of RNA sequencing data showing the differentially expressed genes (DEGs) in CAR, CAR-15 and CAR-21 NK cells (n = 4 donors). **B** The enriched pathways of DEGs in CAR-21 NK cells *vs* CAR NK cells and CAR-21 NK cells *vs* CAR-15 NK cells by GOBP and KEGG enrichment analysis (n = 4 donors). The size of the dots corresponded to the count of genes, and the color of the dots corresponded to the *p value*. **C**–**E** Violin plots showing the fragments per kilobase of exon model per million mapped fragments (FPKM) of cytotoxicity-, metabolic-, and interlukin-21 signaling-related genes in CAR, CAR-15, and CAR-21 NK cell groups (n = 4 donors). **F** GSEA plots showing enrichment in glycolysis pathways in CAR-21 NK cells compared with CAR NK cells and CAR-15 NK cells (n = 4 donors). **G** The extracellular acidification rate (ECAR) was measured in real time in an XFe96 analyzer after injection of rotenone/antimycin (Rot/AA) and 2-deoxy-D-glucose (2-DG) (n = 3 donors).

To investigate the impact of IL-21 expression on the transcriptomic and signaling pathway responses of CAR-NK cells, we performed RNA-sequencing studies of freshly sorted CAR-21 NK cells *vs* CAR and CAR-15 NK cells after co-culture with Raji cells for 8 h. With the threshold of log2-fold change > 1 and Q < 0.05, CAR-21 NK cell group exhibited about 70 Deferentially Expressed Genes (DEGs) with CAR and CAR-15 NK cell groups, with only two DEGs between CAR and CAR-15 NK cells groups (Fig. 4A). The GO enrichment analysis revealed that DEGs in the terms of immune response, cytokine-mediated signaling pathway and kinase activity (Fig. 4B) were the most significant in the function of IL-21(*P* < 0.05). By the KEGG pathway enrichment analysis, pathways related to cytokine-cytokine receptor interaction and JAK-STAT signaling pathway were found to be meaningful targets in the future mechanism research (Fig. 4B). IL-21 co-expressing led to upregulated of genes related to cytotoxicity (eg, *GZMB*, *GZMA*, *TNFRSF8*, *IFNG*, *PRF1*, *CD244*, and *FCGR3A*) (Fig. 4C), metabolic (eg, *MYC*, *HK2*, *PKM*, *SLC2A1*) (Fig. 4D), as well as cytokine signaling (eg, *CEBPD*, *STAT3*, and *SLEE*) (Fig. 4E). Gene set enrichment analysis (GSEA) supported enrichment of pathways involved in glycolysis (Fig. 4F), mTORC1, IL-2/STAT5, and IL-6/JAK/STAT3 signaling, TNFA signaling via NFKB, as well as in those related to inflammatory immune responses (Supplemental Fig. 2). We than used Seahorse assays to measure the glycolytic potential and mitochondrial metabolic of the different CAR-NK cell groups alone and cultured for 24 h with Raji cells. In the absence of Raji cells, CAR-21 NK cells showed the greatest glucose consumption and had higher oxygen consumption rate compared with CAR-15 NK cells (Fig. 4G and Supplemental Fig. 3). When co-cultured with Raji cells, CAR-21 NK cells still showed higher glucose consumption compared to CAR-15 NK cells with a comparable oxygen consumption rate (Supplemental Fig. 3), which parallels the transcription profile. These results align with our previous observation and suggest that IL-21 expression sustaining a cell state with enhanced cytotoxicity and effector function, which is engaged with metabolic reprogramming toward glycolysis.

### Co-expressing IL-21 improves function and cytotoxicity of CAR-NK cells

**Fig. 5:**
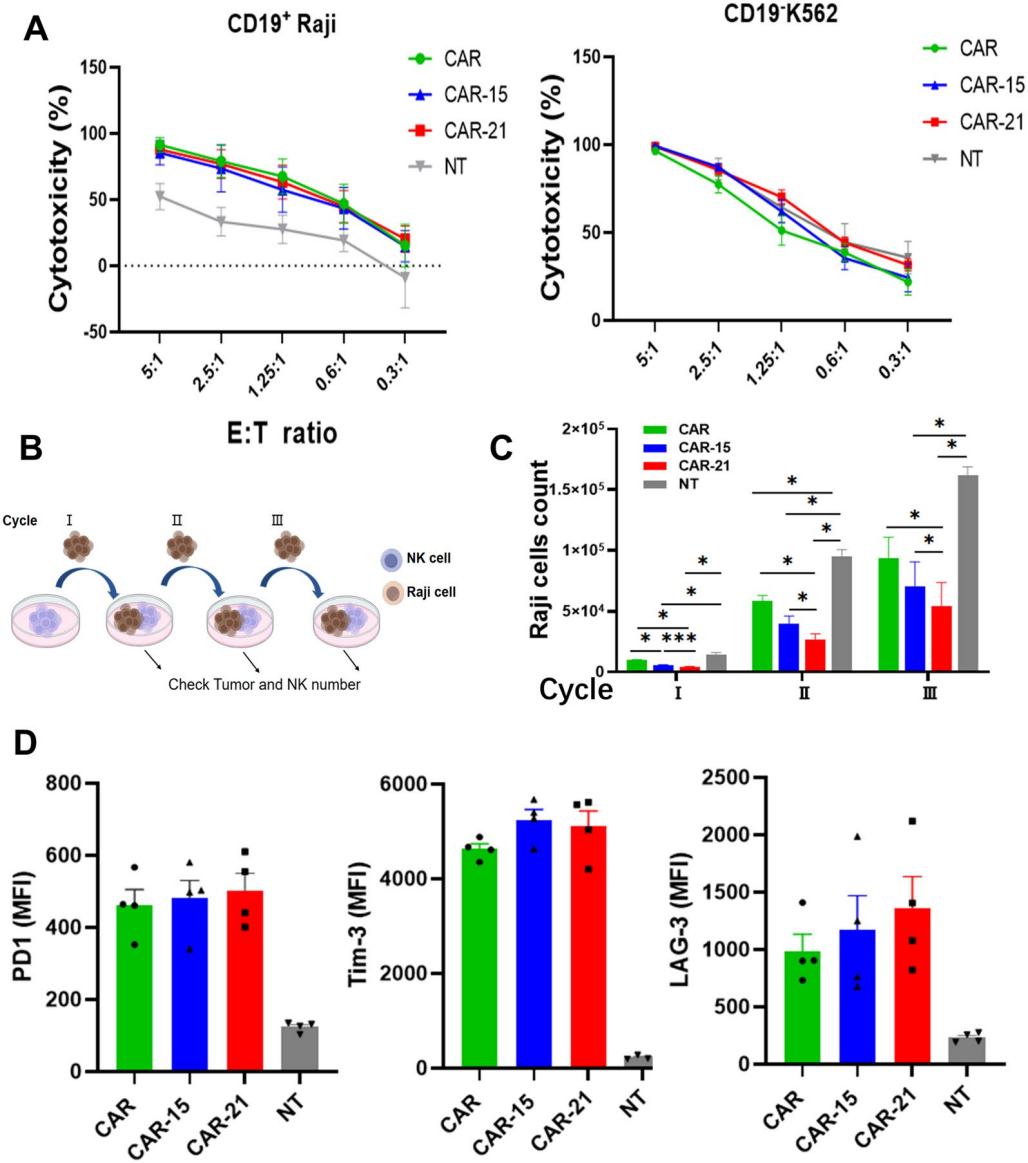
Cytotoxic capacity of CAR-NK cells co-expressing IL-21. **A** Cytotoxicity of NT, CAR, CAR-15 and CAR-21 NK cells against CD19^+^ Raji and CD19^−^ K562 targets expressing firefly luciferase at different effector-to-target (E: T) ratio, as measured by luciferase-based cytotoxicity assay (n = 4 donors). The bars represent mean values with standard error. **B** The schematic of the repetitive tumor stimulation experiment. **C** Testing for killing effect on Raji-GL cells in repetitive co-culturing experiment by flow cytometric cytotoxicity assay (n = 3 donors). **D** The PD-1, Tim-3, and LAG-3 expression of NT, CAR, CAR-15 and CAR-21 NK cells after three rounds of co-culturing with Raji cells in the absence of IL-2 was measured by flow cytometry (n = 4 donors).

The antigen-specific cytotoxic capacity of CAR-NK cells was investigated (Fig. 5A). NT, CAR, CAR-15, and CAR-21 NK cells were co-incubated with Raji-GL and K562-GL cells for 8 h at different efficacy target ratio. The results showed that across all efficacy target ratio, CAR-transduced NK cells exerted superior killing of Raji cells compared to NT NK cells, while CAR-transduced NK cells were equally efficient as NT NK cells in killing K562 targets, indicating that the enhanced killing of CD19 targets by transduced cells is mediated by the CAR receptor and not related to a non-specific enhancement in NK cytotoxicity. In repetitive multi-round co-culture experiments with Raji-GL cells (Fig. 5B), CAR-21 NK cells showed the highest Raji cells elimination at each round of co-cultures (Fig. 5C). We then characterized the exhaustion of NK cells in the repetitive co-culture assay. We found that the exhaustion related markers PD-1, Tim-3 and LAG-3 expression was comparable in CAR-21 NK cell group with CAR and CAR-15 NK cell groups after the three rounds co-culture assay with Raji cells (Fig. 5D). The transcription factors expression of CAR-NK cells was also monitored (Supplemental Fig. 4). No differences in T-bet, EOMES, and GATA3 expression was observed among the CAR-NK cell groups, but expression of these transcription factors was modestly increased after 4 h co-culturing with Raji cells. These findings demonstrated that IL-21 co-expression confers superior anti-tumor effect against lymphoma of CAR-NK cells without functional exhaustion.

### Co-expressing IL-21 promoted CAR-NK cell degranulation and cytotoxicity cytokine production

**Fig. 6:**
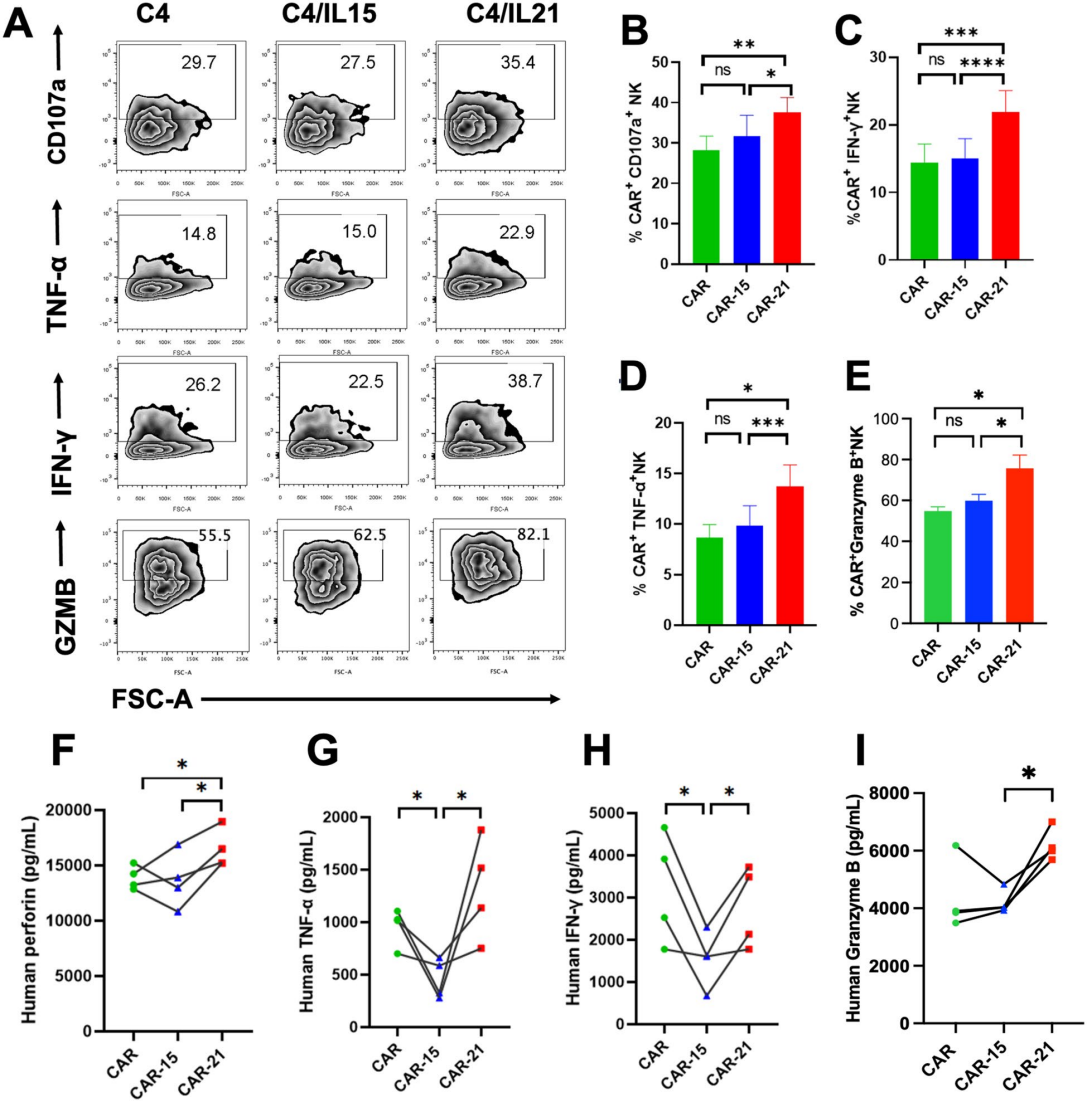
Functionality of CAR-NK cells co-expressing IL-21. **A** CAR-NK cells were co-cultured with CD19^+^ Raji cells for 4 h and harvested for analysis of the surface expression of CD107a and intracellular expression of IFN-γ, TNF-α and Granzyme B. FACS plot showed one representative data from 10 ~ 15 healthy donors. **B**–**E** Summary data of the expression of CD107a, IFN-γ, TNF-α and Granzyme B on CAR-NK cells (n = 10 ~ 15 donors). **F**–**I** The supernatant perforin, IFN-γ, TNF-α and Granzyme B levels after 24 h co-culture of CAR-NK cells with Raji were measured by ELISA (n = 4 donors). ns not significant, * *P* < 0.05, ** *P* < 0.01, *** P < 0.001, **** *P* < 0.0001.

To understand the function mechanism of CAR-21 NK cells targeting Raji cells, the roles for degranulation and lysis of Raji cells were evaluated by ICS and ELISA assays. Significantly, higher CD107a, IFN-γ, TNF-α and Granzyme B expression were found in CAR-21 NK cell group compared to CAR and CAR-15 NK cell groups, while equally higher CD107a, IFN-γ, TNF-α and Granzyme B expression were found in CAR-15 NK cell group compared with CAR NK cell group when co-cultured with Raji cells for four hours (Fig. 6A–E). Additionally, the secretion cytokines were assessed in 24 h co-culture of Raji cells with CAR, CAR-15 and CAR-21 NK cell groups by ELISA (Fig. 6F–I). The level of perforin, IFN-γ, TNF-α and Granzyme B secretions was observed relatively higher from CAR-21 and CAR NK cell groups than those from CAR-15 NK cell group, while no notable difference was observed in those expression level among the CAR-21 and CAR NK cell groups. These results suggested that IL-21 co-expressing enhanced CAR-NK cells to produce the higher levels of various cytokines, which played the important roles attributing to the cytotoxicity effect on Raji cells.

### CAR-NK cells co-expressing IL-21displayed superior in vivo anti-tumor efficacy

**Fig. 7:**
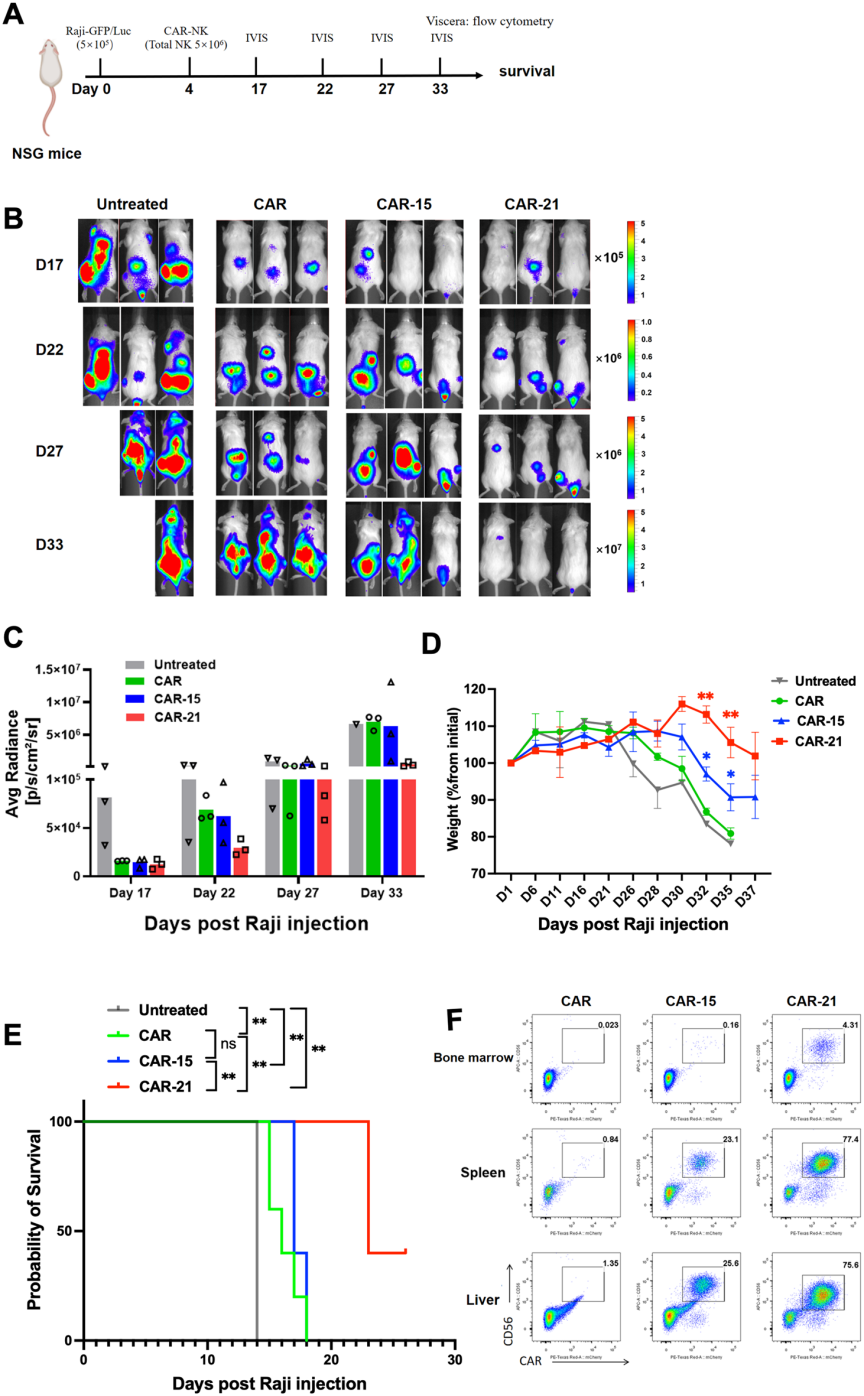
Antitumor efficacy of CAR-NK cells co-expressing IL-21 in vivo. **A** Schema of in vivo studies. Briefly, NSG mice received 5 × 10^5^ Raji cells IV followed by 5 × 10^6^ NK cells IV injection on day 4, containing 85% to 95% CAR positive NK cells. **B** Tumor burden was monitored using IVIS, and representative data of the bioluminescence mice are show. **C** The average radiance is shown for the untreated group, as well as CAR, CAR-15 and CAR-21 NK cell treatment groups of mice (n = 3). **D** Body weight of mice was monitored every 2 to 5 days (n = 3); asterisks depict statistical significance for the comparison. Red asterisks: CAR-21 vs CAR-15 NK; blue asterisks: CAR-15 vs CAR NK; one-way ANOVA with Bonferroni correction. **E** Kaplan-Meier survival curve of mice receiving tumor only and CAR-NK cells treatment (n = 5); log rank test. Data was collected from another independent experiments. **F** FACS showing the percentage of CAR^+^ NK cells (CD56^+^ mCherry^+^) present in organs from mice treated with CAR, CAR-15, and CAR-21 NK cells at day 33.

We next examined the anti-tumor activity of CAR-21 NK cells in vivo. NSG mice were infused IV GFP and Luciferase-expressing Raji cells and a single injection of CAR, CAR-15 or CAR-21 NK cells (Fig. 7A). Tumor burden assessed by IVIS in CAR-21 NK-recipient mice was lower than that in CAR and CAR-15 NK -recipient mice as well as untreated mice (Fig. 7B, C), suggesting that CAR-21 NK cells exhibited stronger anti-tumor activity than CAR and CAR-15 NK cells. Compared with CAR-NK cell groups treated mice, untreated tumor-bearing mice showed a significant reduction of body weight from day 26 (about 10% weight loss) post tumor injection (Fig. 7D), suggesting tumor progression in these mice. Comparing in the mean survival of each treatment group in the Raji lymphoma mouse model, the results were CAR-21 > CAR-15 > CAR > untreated group, in which the survival time of CAR-NK cell treatment groups was significantly longer than that of untreated group (*P* < 0.01), and the survival time of CAR-21 group was significantly longer than that of CAR and CAR-15 groups (*P* < 0.01) (Fig. 7E). All surviving animals were sacrificed at day 33 and their organs examined for evidence of lymphoma and CAR-NK cells. While all animals had no evidence of tumor in their organs, those treated with CAR-21 NK cells had more percentage of CAR^+^NK cells in their spleen, liver, and bone marrow (Fig. 7F), this result may be associated with improved NK cell persistence in mice that received CAR-21 NK cells. Once the mice exhibit bilateral hind limb paralysis or weight loss exceeding 20% of their initial body weight, mice were succumbed to tumor, as demonstrated by FACS (Supplemental Fig. 5). These data demonstrated that CAR-21 NK cells provide enhanced tumor control in vivo.

## Discussion

In this study, we demonstrate that arming CAR-NK cells with IL-21 enhances their effector function and provides long-lasting anti-tumor activity through metabolic reprogramming, compared to CAR-NK cells modified to express with IL-15.

To improve the functionality of NK cells, several modifications have been designed to tackle specific challenges. Cytokines can be utilized to further augment the potency and in vivo persistence of NK cells in combating cancer [28–30]. However, the optimal cytokine support required after adaptive transfer to promote NK cell expansion and persistence remains unclear. IL-21 has emerged as the latest discovery within the γ chain cytokine family, known for its crucial effects on both innate and adaptive immunity [12, 31]. Several studies have reported the potent cytotoxic effect of IL-21 against cancer cells. T cell receptor-engineered T cells (TCR-T) modified with IL-21 receptors demonstrated enhanced proliferation upon activation and superior anti-tumor function both in vitro and in vivo compared to conventional TCR-T against hepatocellular carcinoma [32]. The administration of recombinant IL-21 has been shown to rejuvenate exhausted NK cells in both animal models and human cancer patients [22]. Based on these findings, several clinical trials are currently underway.

IL-15 has emerged as a central subject of translational clinical research due to its ability to augment the cytotoxic activity, proliferation, and longevity of NK cells [33, 34]. Several clinical trials involving patients with malignancies have demonstrated that the potential efficacy of systemically administered NK cells that have been genetically modified to express IL-15 and a CAR [8, 9]. However, it has been observed that infusion of CAR-T cells engineered with IL-15 caused severe liver toxicity and GVHD in the xenograft mouse model [35]. Additionally, various studies have indicated that IL-15 signaling may play a role in promoting cancer progression [36, 37]. IL-15 can stimulate the activation of recipient CD8^+^ T cells, which subsequently accelerate the rejection of donor NK cell, thereby limit the efficacy of allogeneic cellular therapy [16–18]. A recent study revealed that NK cells modified to express IL-21 maintained their anti-tumor activity against glioblastoma over time, whereas IL-15 armored NK cells were more prone to exhaustion after multiple tumor re-challenges [25]. However, the pleiotropic effects of IL-21 on CAR-NK cell-mediated tumor rejection remain underexplored. In this study, we reported that engineering CAR-NK cells to express IL-21 resulted in enhanced anti-tumor activity compared to CAR-NK cells expressing IL-15. Using a xenograft mouse model of NK-resistant Raji lymphoma, we demonstrate that IL-21 co-expression improves the in vivo persistence of CAR-NK cells. Additionally, this increased anti-tumor activity was not associated with higher toxicity in these animals. Although the NSG mouse model has limitations for studying CRS, the absence of overt toxicity, such as rapid weight loss and early deaths, support the safety of our approach.

STAT3, a known target of IL-21 [38, 39], has been implicated in the regulation of *CEBPD* expression [25]. Our data also support a potential role of STAT3 in the regulation of *CEBPD* by IL-21 in CAR-NK cells. We attributed this gain of function to IL-21-driven mTORC1 signaling, resulting in a consequent increase in glycolytic activity. These findings are consistent with recent report that engineering NK cells to express IL-21 enhances their metabolism and anti-tumor activity via *CEBPD* [25]. Our results demonstrated that engineered CAR-NK cells expressing IL-21 were associated with the upregulation of metabolic-related genes *PKM* and *SLC2A1*, as well as cytotoxicity-related genes *GZMB*, *GMZA*, *IFNG*, *TNFRSF8*, *PRF1*. This suggests that constructing IL-21-secreting CAR-NK cells enhances their metabolic fitness and endows them with superior anti-tumor function.

As demonstrated by Shanley et al., IL-21-armed NK cells exhibit potent antitumor activity [25]. However, numerous tumor types, including Raji cells, demonstrate resistance to NK cell-mediated cytotoxicity through various mechanisms, particularly the downregulation or absence of ligands for NK-activating receptors [7, 28, 40]. In such cases, the development of IL-21-secreting CAR-NK cells may provide enhanced mechanistic synergy and overcome tumor resistance mechanism. While our findings underscore the significant potential of IL-21-expressing CAR-NK cells to enhance antitumor activity, two critical limitations persist that must be addressed to fully realize their therapeutic promise. One major limitation is the potential for increased toxicity and CRS associated with IL-21 expression. IL-21 can enhance the activity and persistence of CAR-NK cells, but it may also lead to excessive cytokine production, which requires evaluation in humanized mouse models to assess the in vivo toxicity of CAR-IL-21 NK cells. Although IL-21 drives metabolic reprogramming towards glycolysis in CAR-NK cells, the precise regulatory mechanisms underlying this process remain unclear. Further mechanistic studies are therefore warranted to elucidate these pathways and optimize the metabolic fitness of CAR-NK cells for enhanced therapeutic efficacy.

In conclusion, we demonstrated that IL-21 engineered, CD19-specific CAR-NK cells showed enhanced anti-tumor efficacy against CD19^+^ lymphoma cells both in vitro and in vivo settings, as well as increased persistence in a mouse xenograft model of lymphoma compared to IL-15 engineered CAR-NK cells. This highlights their potential as a promising therapeutic approach for patients with relapse or refractory B cell malignancies. Beyond CAR-NK cells, the incorporation of IL-21 into CAR-T and CAR-NKT cell therapies has shown significant potential in enhancing the efficacy of these treatments [32, 41]. By improving proliferation, tumor infiltration, cytokine secretion, and metabolic reprogramming, IL-21 can help address some of the major challenges associated with CAR-based therapies. Future research should focus on optimizing the delivery and expression of IL-21, as well as exploring its synergistic effects with other immunomodulatory agents.

## Supplementary Information

**Supplementary materials 1.**

## Data Availability

No datasets were generated or analysed during the current study.

## Abbreviations

NK: Natural killer
IL-21: Interleukin-21
CAR: Chimeric antigen receptor
FDA: Food and drug administration
CRS: Cytokine release syndrome
ICANS: Immune effector cell-associated neurotoxicity syndrome
GVHD: Graft-versus-host disease
PBMCs: Peripheral blood mononuclear cells
scFv: Single-chain variable fragment
CDRs: Complementarity-determining regions
