# Supplementary material for: Interleukin-21 engineering enhances CD19-specific CAR-NK cell activity against B-cell lymphoma via enriched metabolic pathways
Source: Exp Hematol Oncol. 2025 Apr 2;14:51. doi: 10.1186/s40164-025-00639-2 (PMC11967061; doi:10.1186/s40164-025-00639-2)
Supplement: Supplementary file 1 — Supplementary materials 1. [file 40164_2025_639_MOESM1_ESM.docx]

**Supplemental Data**

**Supplemental Methods**

**Cell lines**

CD19^+^ Raji (human Burkitt’s lymphoma cell line) and CD19^-^ K562 (human myelogenous leukemia cell line) were obtained from the American Type Culture Collection. To establish the K562-FFLuc and Raji-FFLuc cells, K562 and Raji cells were transduced with a lentiviral supernatant encoding Firefly Luciferase and GFP. K562-based feeder cells were electroporationally transduced to co-express 4-1BBL and membrane-bound IL-15 (mbIL-15) using the Sleeping Beauty transposon system, which was kindly provided by Kaibo Yang (Southern Medical University Nanfang Hospital). All these cell lines were cultured in Roswell Park Memorial Institute (RPMI) 1640 medium supplemented with 10% heat-inactivated fetal bovine serum (FBS, Gibco), and 1% penicillin/streptomycin (Invitrogen) at 37°C in a humidified 5% CO_2_ incubator. HEK-293T/17 cells purchased from the National Collection of Authenticated Cell Cultures (Shanghai, China) and were maintained in Dulbecco modified Eagle medium (DMEM, Gibco) with 10% FBS and 1% penicillin/streptomycin (Invitrogen).

**Flow cytometry**

For surface markers, cells were stained at room temperature or 4 ℃ for 15 to 20 minutes using fluorophore-conjugated antibodies. In this study, the following antibodies purchased from BioLegend were used: FITC/PerCP/PE-Cy7-conjugated CD3 (clone OKT3), APC/BV421-conjugated CD56 (clone NACM), BV510-conjugated CD16 (clone 3G8), PE-conjugated CD25 (clone BC96), FITC-conjugated CD69 (clone FN50), APC-conjugated NKG2D (clone 1D11), BV510-conjugated PD-1 (clone EH12.2H7), Pacific blue-conjugated Tim-3 (clone F38-2E2LAG3), PE-Cy7-conjucted LAG-3 (clone 11C3C65), APC-conjugated NKG2A (clone S19005E), APC-conjugated T-bet (clone O4-46), BV421-conjugated GATA3 (clone L50-823), FITC-conjugated EOMES (clone X4-83), PE-Cy7-conjugated CD107a (clone H4A3), BV510-conjugated TNF-α (clone MAb11), PE-conjugated IFN-γ (clone 4S.B3). Fluorochrome-labeled monoclonal antibody LIVE/DEAD Fixable Dead Cell Staining (APC-Cy7, L34992, Thermo, USA) was used to exclude dead cells. All samples were acquired using a BD LSRFortessa X-20 flow cytometer following standard protocols. Data analysis was performed on FlowJo V10 software.

**Cell viability and proliferation detection**

CAR-NK cell viability was assessed using the LIVE/DEAD Fixable Dead Cell Staining kit, and proliferation was quantified by flow cytometry using the Flow Cytometry Absolute Counting Beads kit, following the manufacturer’s protocol (CountBright Absolute Counting Beads, C36950, Invitrogen, USA) (n = 4).

**Immunophenotype analysis**

The phenotypes of NT-NK, CAR, CAR-15 and CAR-21 NK cell groups, including specific activation markers (CD25, CD69 and NKG2D), inhibition markers (NKG2A) and exhaustion markers (PD-1, LAG-3 and TIM-3), transcription factors (EOMES, T-bet, GATA3) were detected by flow cytometry using specific antibodies. CD16 and CD56 were used to identify the NK cell subsets, including CD56^bright^ CD16^+^, CD56^bright^ CD16^-^, CD56^dim^ CD16^+^ and CD56^dim^ CD16^-^ cells.

**RNA-seq analysis**

Raji cells (3×10^6^) were seeded into 6-well culture plates, followed by the addition of CAR, CAR-15 or CAR-21 NK cells at an effector-to-target ratio of 1:1(with effectors defined as mCherry^+^ cells). After 8 hours co-culture, NK cells (n =4) were purified using a human NK isolation kit (Miltenyi Biotec) according to the manufacturer’s instructions. Following RNA extraction with TRIzol reagent (Invitrogen) and quality inspection of purify and integrity by NanoPhotometer and Agilent 2100 Bioanalyzer, the qualified RNA was enriched using oligo (dT)-coated magnetic beads, and fragmented and then used as the template for complementary DNA (cDNA) synthesis. The quality of the cDNA library was assessed using an Agilent 2100 Bioanalyzer, and sequencing was performed on the DNBSEQ platform (BIG, Guangzhou, China). Raw sequencing data underwent quality control and adapter trimming using fastp software (<https://github.com/OpenGene/fastp>). The trimmed reads were then mapped to the human genome (hg38) using HISAT2 (<http://daehwankimlab.github.io/hisat2/>) with default parameters. Reads counts for each gene were obtained using Feature Counts (<http://subread.sourceforge.net/>). Gene expression abundance estimated by normalized to the number of fragment per kilobase of exon model per million mapped reads (FPKMs) was obtained using edgeR package. The Principal Component Analysis (PCA), Differentially Express Genes (DEGs), Gene Ontology (GO), Kyoto Encyclopedia of Genes and Genomes (KEGG) pathway enrichment analysis and Gene Set Enrichment Analysis (GSEA) were performed.

**Enzyme-linked immunoscorbent cytokine assay (ELISA) and apoptosis assay**

The levels of IL-21 and IL-15 were measured using human IL-21 ELISA Kit (MultiSciences) and human IL-15 ELISA Kit (R&D), respectively, according to the manufacturer’s instructions. NK cells were infected with different CAR vectors in 96-well plates in triplicate wells using NK cell medium without cytokine and cultured for 24, 48 and 72 hours, respectively. Every 24 hours, half of the supernatant in each well was gently removed and then replaced with an equivalent volume of fresh NK cell medium. Subsequently, supernatants were collected for the detection of IL-15 and IL-21, while cells were harvested for viability, proliferation and apoptosis assays. The release of perforin, IFN-γ, TNF-α (ABclone) and Granzyme (MultiSciences) in CAR-NK cells co-cultured with Raji cells at a 1:1 ratio for 24 hours was also assessed by ELISA.

**Supplemental Figures**

Supplemental Figure 1


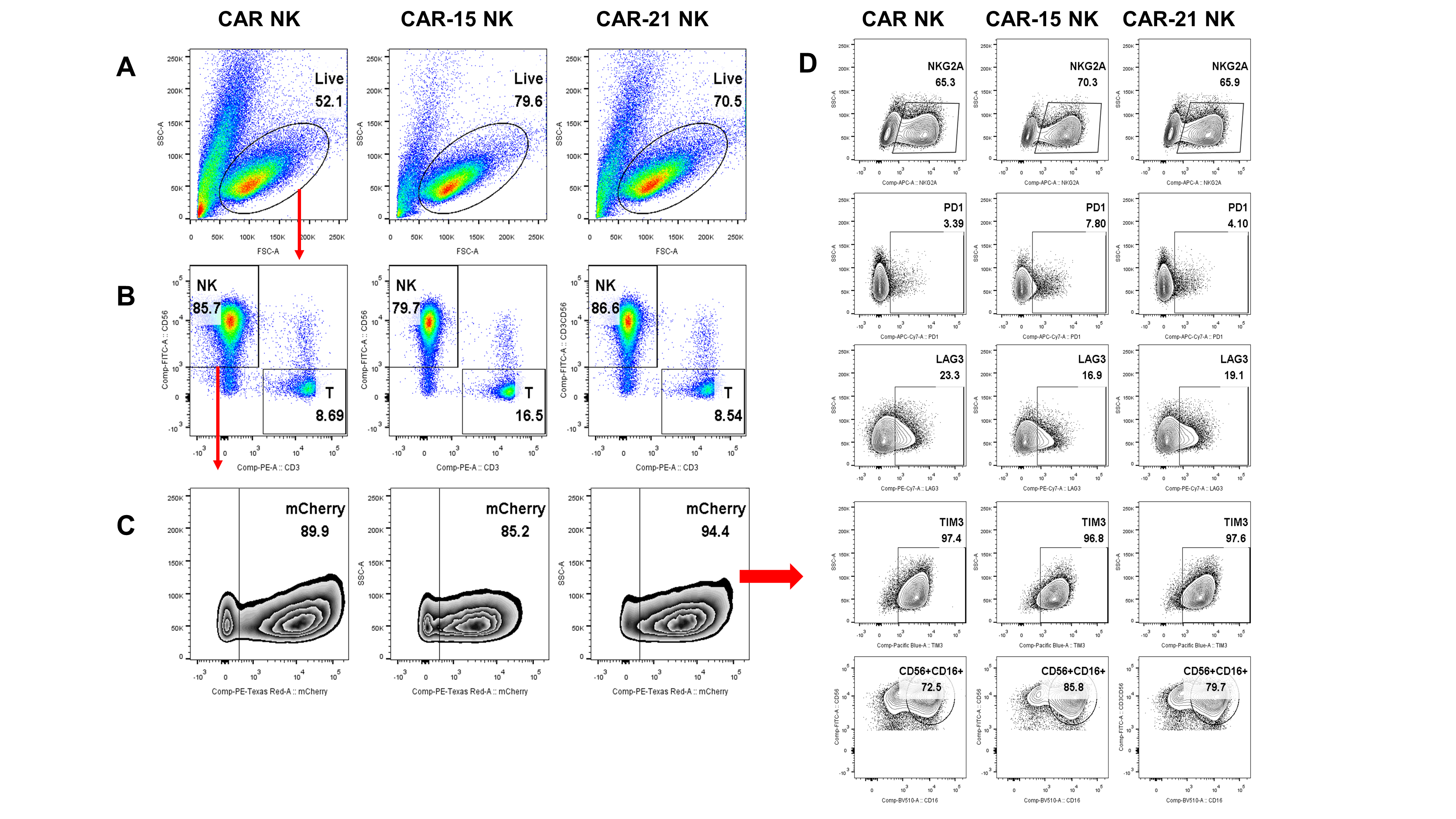


Supplemental Figure 1. The transduction efficiencies of CAR, CAR-15 and CAR-21 NK cells and their phenotype signatures which used to treat Raji lymphoma mouse model

Supplemental Figure 2

**
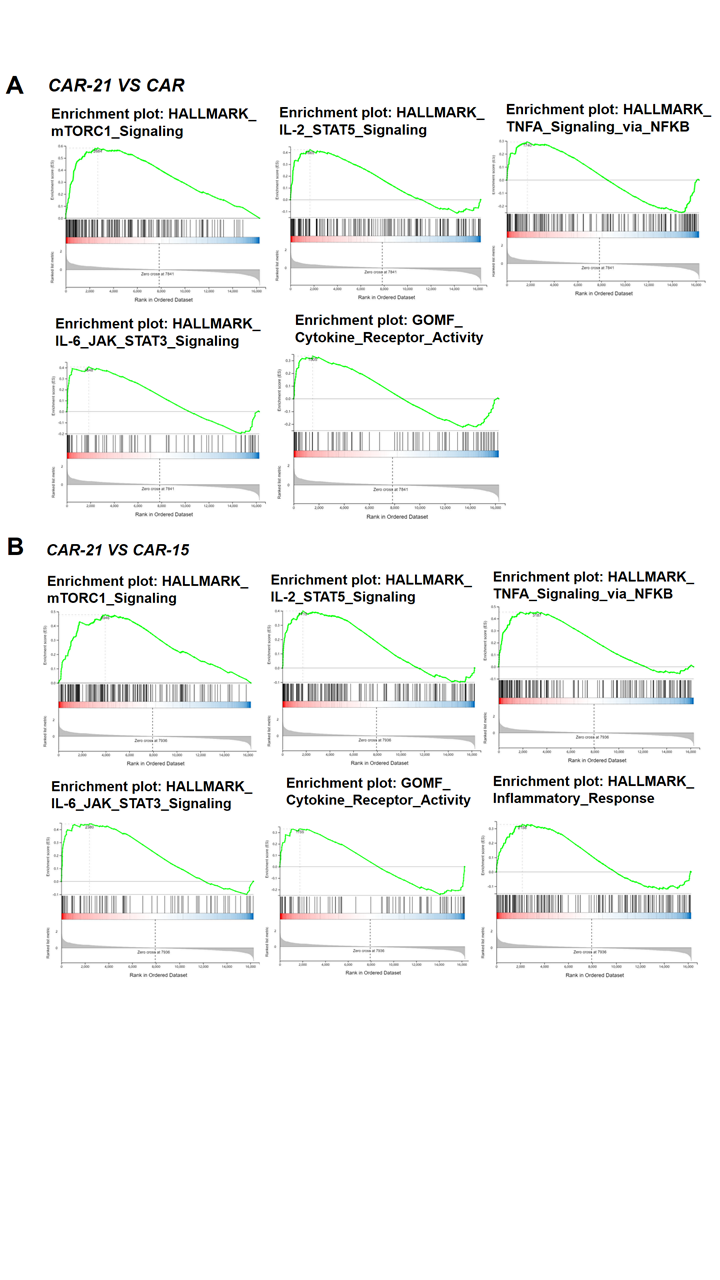
**

Supplemental Figure 2**.** Related pathways identified by GSEA pathway enrichment

Supplemental Figure 3


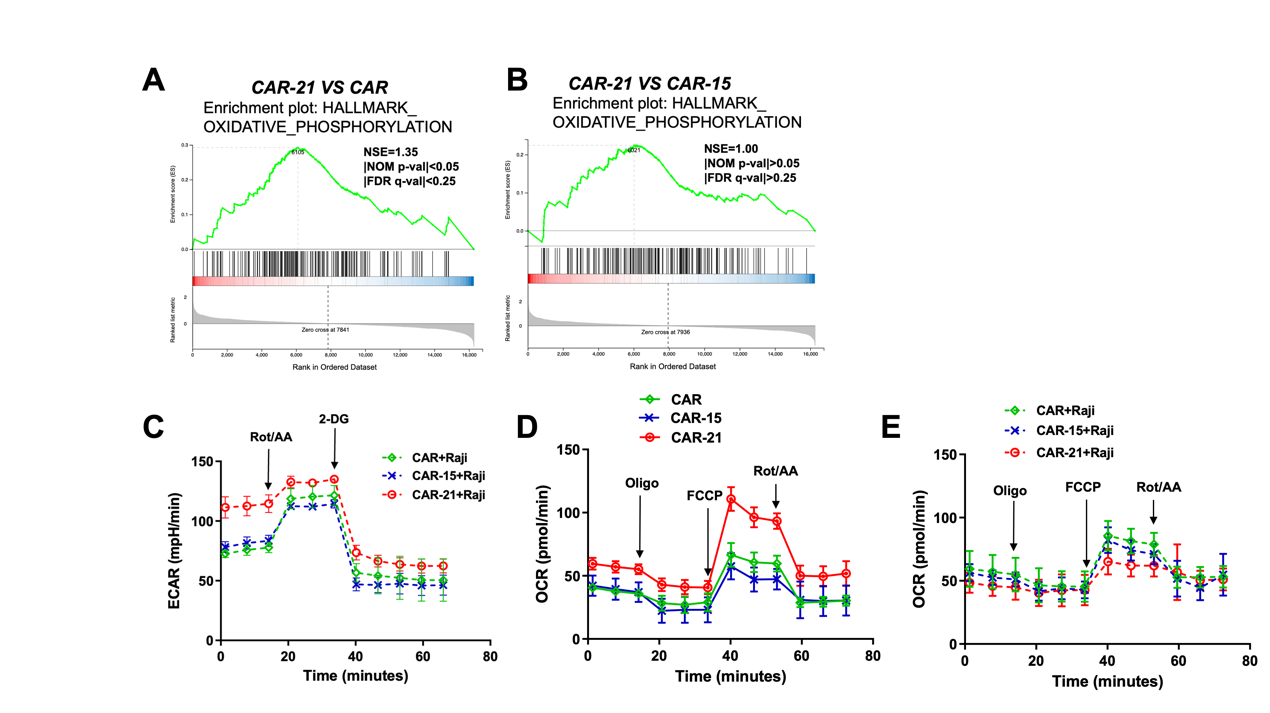


Supplemental Figure 3. Metabolic analysis of CAR-NK cells.

(A, B) GSEA plots showing enrichment in Oxidative Phosphorylation pathways in CAR-21 NK cells compared with CAR NK cells and CAR-15 NK cells (n = 4 donors).

(C) ECAR was calculated for CAR-NK cells co-cultured with Raji targets for 24 hours and subsequently purified and treated with 0.5 μM rotenone/antimycin A and 50 mM 2-Deoxyglucose (2-DG) (n = 3). OCR was calculated for CAR-NK cells alone (D) or co-cultured with Raji targets for 24 hours and subsequently purified (E) and treated with 1.5 μM oligomycin, 1.5 μM carbonyl cyanide-4 (trifluoromethoxy) phenylhydrazone (FCCP) and 0.5 μM rotenone/antimycin A (n = 3).

Supplemental Figure 4

**
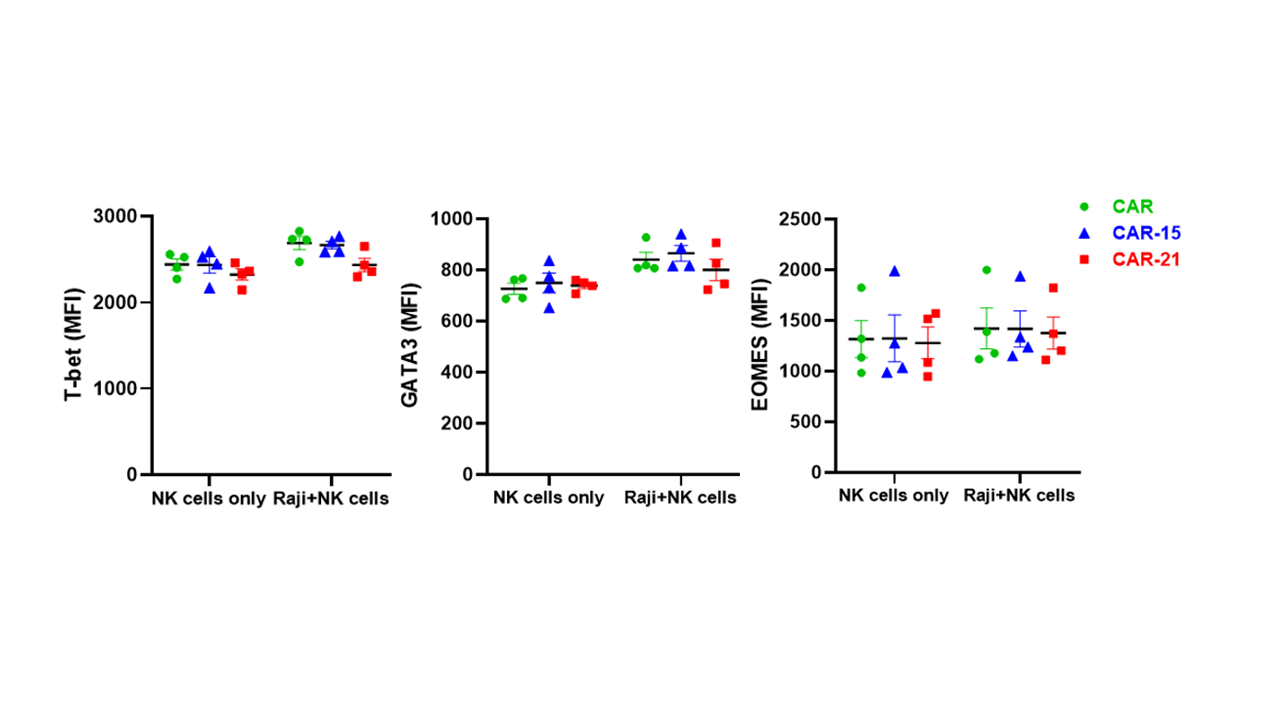
**

Supplemental Figure 4. The transcription factors expression (EMOES, T-bet, GATA3) of CAR-NK cells stimulated with or without Raji cells was measured by flow cytometry (n=4)

Supplemental Figure 5


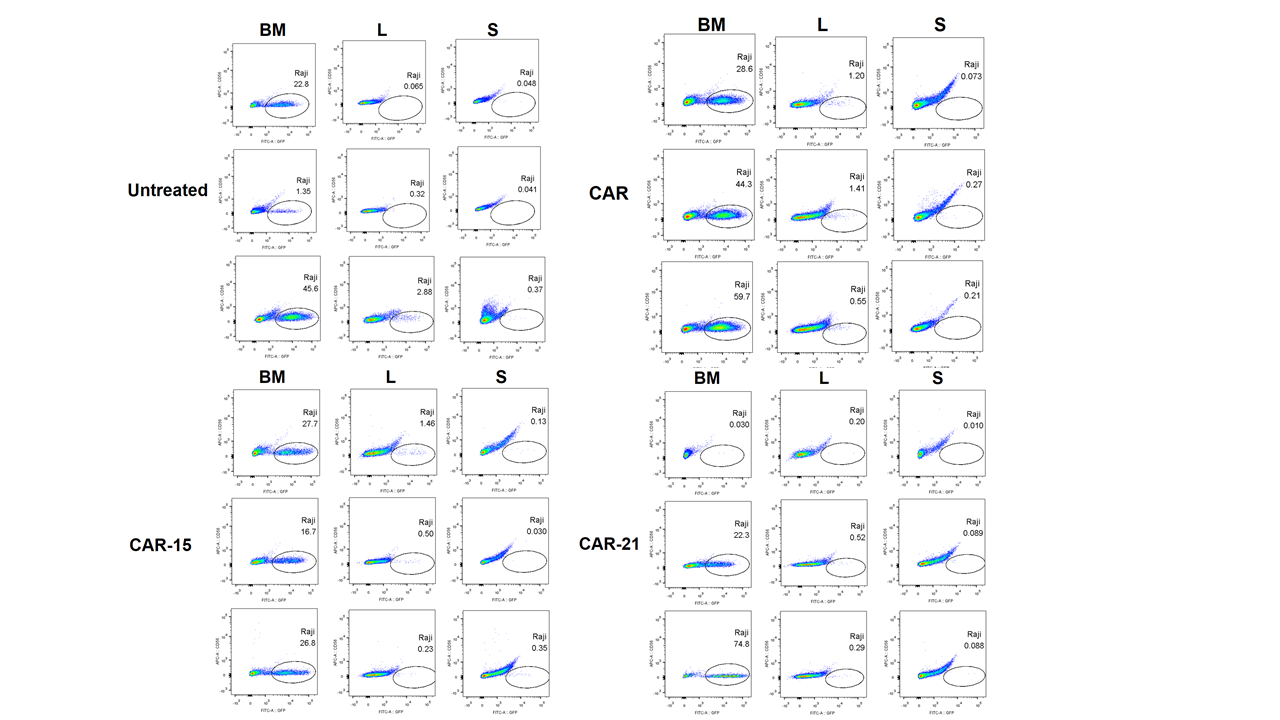


Supplemental Figure 5. The proportions of Raji cells in the liver, spleen, and bone marrow of mice treated with CAR, CAR-15, and CAR-21 NK cells, as well as untreated treated control group were analyzed using flow cytometry
